# Involvement of the epidermal growth factor receptor in IL‐13–mediated corticosteroid‐resistant airway inflammation

**DOI:** 10.1111/cea.13591

**Published:** 2020-03-09

**Authors:** Elizabeth R. Davies, Jeanne‐Marie Perotin, Joanne F.C. Kelly, Ratko Djukanovic, Donna E. Davies, Hans Michael Haitchi, the U‐BIOPRED Study Group

**Affiliations:** ^1^ Brooke Laboratories Clinical and Experimental Sciences Faculty of Medicine University of Southampton Southampton UK; ^2^ National Institute for Health Research (NIHR) Southampton Biomedical Research Centre University Hospital Southampton NHS Foundation Trust Southampton UK; ^3^ Institute for Life Sciences University of Southampton Southampton UK

**Keywords:** animal models, asthma, basic mechanisms, corticosteroid‐refractory, epithelium, neutrophils

## Abstract

**Background:**

Effective treatment for severe asthma is a significant unmet need. While eosinophilic inflammation caused by type 2 cytokines is responsive to corticosteroid and biologic therapies, many severe asthmatics exhibit corticosteroid‐unresponsive mixed granulocytic inflammation.

**Objective:**

Here, we tested the hypothesis that the pro‐allergic cytokine, IL‐13, can drive both corticosteroid‐sensitive and corticosteroid‐resistant responses.

**Results:**

By integration of in vivo and in vitro models of IL‐13–driven inflammation, we identify a role for the epidermal growth factor receptor (EGFR/ERBB1) as a mediator of corticosteroid‐unresponsive inflammation and bronchial hyperresponsiveness driven by IL‐13. Topological data analysis using human epithelial transcriptomic data from the U‐BIOPRED cohort identified severe asthma groups with features consistent with the presence of IL‐13 and EGFR/ERBB activation, with involvement of distinct EGFR ligands. Our data suggest that IL–13 may play a dual role in severe asthma: on the one hand driving pathologic corticosteroid‐refractory mixed granulocytic inflammation, but on the other hand underpinning beneficial epithelial repair responses, which may confound responses in clinical trials.

**Conclusion and Clinical Relevance:**

Detailed dissection of those molecular pathways that are downstream of IL‐13 and utilize the ERBB receptor and ligand family to drive corticosteroid‐refractory inflammation should enhance the development of new treatments that target this sub‐phenotype(s) of severe asthma, where there is an unmet need.

## 1 INTRODUCTION

Asthma is a heterogeneous disease characterized by a diverse profile of symptoms, severity and responses to medications. In mild‐to‐moderate asthma, treatment with inhaled corticosteroids can significantly reduce inflammation and control symptoms. However, in patients with more severe disease, symptoms can persist despite receiving standard of care treatment, including antibody‐based biologics.^1^ This subgroup has been defined as “severe refractory” asthma.^2^, ^3^ Up to 10% of the asthmatic population are classed as severe; they have higher rates of asthma exacerbations, increased morbidity and account for a disproportionate use of healthcare resources, accounting for more than 60% of the economic burden.^4^ Much work has been done to advance the clinical understanding of severe refractory asthma, but there still remains a clear unmet clinical need.^4^

The recent focus on disease heterogeneity has raised the concept that asthma consists of multiple phenotypes with distinct underlying mechanisms (endotypes).^4^, ^5^, ^6^, ^7^ Initially, the focus was on identification of subgroups based on clinical presentation including exacerbations, persistent symptoms and reduced lung function. However, with the advent of 'omic technologies (transcriptomics, proteomics, metabolomics, lipidomics) and the recruitment of large patient cohorts,^8^, ^9^, ^10^, ^11^ molecular and biological processes can now be evaluated with the aim of identifying distinct endotypes. A key phenotypic characteristic that is used to define asthma subgroups is the presence or absence of biomarkers of type‐2 inflammation.^4^ Typical type‐2 biomarkers include bronchial epithelial gene expression of *POSTN* (Periostin), *CLCA1* (Chloride Channel Accessory 1) and *SERPINB2* (Serpin Family B Member 2),^12^ blood and sputum eosinophils,^13^ fractional exhaled nitric oxide (Feno),^14^ serum periostin,^12^, ^15^ serum IgE 13 and type‐2 gene expression (*IL4*, *IL5*, *IL13*) in sputum cells*.* 16 Elevated levels of these biomarkers are frequently associated with the presence of atopy and bronchial hyperresponsiveness (BHR)^17^ and are considered to be sensitive to inhibition by corticosteroids and monoclonal antibodies against IgE, IL‐5 or IL‐5 receptor.^1^, ^12^, ^17^ In contrast, patients who have “non‐type‐2” (also known as “type‐2–low”) severe asthma are frequently characterized by airway neutrophilia, a type‐17 immune signature involving genes such as *CXCL1*, *CXCL2* and *CSF3* 18 and disease that is not effectively treated by inhaled corticosteroids or biologics.^6^

While this broad classification of corticosteroid responsiveness in relation to type‐2 inflammation is a reasonable generalization, studies with dupilumab, an antibody to IL‐4Rα, suggest it is an over‐simplification. Thus, treatment of uncontrolled persistent asthmatic patients with dupilumab as an add‐on therapy increased lung function and reduced severe exacerbations irrespective of baseline eosinophil count.^19^ Subsequent studies confirmed that dupilumab treatment resulted in a reduction in glucocorticoid use and severe exacerbations.^20^, ^21^ As these beneficial effects of dupilumab, which inhibits both IL‐4 and IL‐13 signalling,^22^ were seen in patients who were receiving medium‐to‐high‐dose inhaled corticosteroids plus a long‐acting β2 agonist, this suggests that these asthma patients have persistent type‐2 responses despite inhaled corticosteroid therapy. Consistent with this, it has been reported that airway type‐2 inflammation is not suppressed adequately in approximately half of asthmatic patients treated with inhaled corticosteroids^23^ and that there are many severe asthma patients with mixed eosinophilic and neutrophilic inflammation.^24^

Based on these clinical observations, the aim of this work was to model allergic airway inflammation using a transgenic IL‐13 mouse^25^ and to test the hypothesis that a subset of IL‐13–induced responses are corticosteroid‐unresponsive and contribute to ongoing airway symptoms. We show that IL‐13–induced airway inflammation involves a corticosteroid‐refractory component characterized by pro‐neutrophilic cytokine expression, airway neutrophilia and BHR. IL‐13–driven pro‐neutrophilic cytokine expression was mediated by EGFR activation which was corticosteroid unresponsive. To translate these findings into human disease, we investigated gene epithelial expression in the U‐BIOPRED cohort. Our analyses suggest that IL‐13 may have a dual role, on the one hand driving mixed granulocytic inflammatory responses, but on the other hand underpinning epithelial repair responses. Thus, targeting IL‐13 may have both beneficial and detrimental effects, confounding responses in clinical trials.

## 2 METHODS

### 2.1 Mice

Doxycycline (DOX)‐inducible conditional double‐transgenic mice expressing a (tetO)_7_‐CMV‐*Il13* transgene under the regulation of the line 1 *Club Cell Secretory Protein (Ccsp)* promoter (*Scgb1a1*) have been previously described.^25^ Expression of IL‐13 was induced in 6‐week‐old double‐transgenic (*Ccsp/Il13*) mice by provision of Doxycycline (DOX) (Lab Diet, 5LOS W/625 ppm DOX; TestDiet) in the food ad libitum*.* Single transgenic littermate controls were fed the same diet. Where indicated, *Ccsp/Il13* mice were given daily intraperitoneal injections of 3 mg/kg Dexamethasone (DEX) (Hameln Pharmaceuticals) or 0.1 mg/kg EGFR inhibitor AG1478 (Bio‐Techne) for up to 7 days, while control mice received saline. Experiments followed the 3R (Replacement, Reduction, and Refinement) principles, and experiments were conducted according to the Animal Research: Reporting of In Vivo Experiments (ARRIVE) guidelines^26^ and the local Southampton University ethical committee under project and personal licences from the Home Office, United Kingdom.

### 2.2 Mouse tracheal epithelial cell expansion

Tracheal epithelial cells were isolated and expanded as previously described.^27^ Isolated cells were grown on Transwells^®^ (Corning) in submerged culture to allow formation of tight junctions, determined by trans‐epithelial electrical resistance (TER). Cells were cultured until the TER was >1000 Ω.cm^2^. IL‐13 was induced by addition of 2 µmol/L DOX (Sigma Aldrich) to the culture medium. When required, AG1478 (Sigma Aldrich) and/or Dexamethasone (Hameln) were added to the culture medium, each at a concentration of 10 µmol/L. Apical and basolateral secretions were collected at 48 and 72 hour for KC and CXCL2 protein quantification and cells removed from the transwells in TRIzol and pooled in duplicates for RNA extraction.

### 2.3 Assessment of lung function

Mice were anaesthetized with 100 μL of anaesthetic containing a 4:1:1 mixture of ketamine, acepromazine and xylazine by intraperitoneal injection. A FlexiVent system (SCIREQ) was used to assess lung function in the form of airway resistance (R) after aerosolized methacholine challenge to provide a measure of BHR, according to the manufacturer's instructions. Airway resistance was measured by forced oscillation technique, with increasing values indicating bronchoconstriction of the lungs. BHR measurements were obtained from individual animals using increasing stepwise concentrations of 0, 2.5, 5 and 10 mg/mL methacholine (Sigma Aldrich).

### 2.4 Inflammatory cell counts

Bronchoalveolar lavage (BALF) samples were collected by washing the lungs three times with 800 μL sterile PBS. The total volume of the combined fluids was measured and then centrifuged at 300 *g* for 5 minutes. The BALF supernatants were frozen for cytokine analysis. Red blood cells were lysed from the cell pellets, which were subsequently resuspended in 300 μL PBS. Cells were counted, and 100 000 cells were loaded into a cytospin funnel and centrifuged at 300 *g* for 5 minutes on to a glass slide. Slides were air‐dried, and the cells were stained using a Diff‐Quick stain (Siemens). The different inflammatory cell types were counted to a total of 300 cells and expressed as the differential cell count in cells/mL of BALF.

### 2.5 RNA extraction and analysis

Lung tissue was stored in RNAlater (Life Technologies) before homogenization in Qiazol^®^ Reagent and RNA isolated on columns from miRNeasy Kits (Qiagen). mTEC RNA was isolated via TRIzol^®^ extraction (Life Technologies) using standard protocols, and genomic DNA contamination was removed by digestion with DNase (Life Technologies). mRNA expression was measured by reverse transcription quantitative PCR. First‐strand cDNA was generated by reverse transcription using the NanoScript2 cDNA synthesis kit (PrimerDesign). qPCR was performed using a CFX96 qPCR machine (Bio‐Rad) for 40 cycles at 95°C for 5 seconds and 60°C for 20 seconds (fast protocol) or 40 cycles at 95°C for 15 seconds and 60°C for 60 seconds (standard protocol). Each reaction contained either Expression Master Mix (Life Technologies) or Precision Master Mix (PrimerDesign) and a Taqman primer/probeset for one of the following targets: *Il‐13* Mm00434204_m1; *Il‐17a Mm00439618_m1, Cxcl1/Kc* Mm04207460_m1; *Ccl11/Eotaxin* Mm00441238_m1; *Muc5ac* Mm01276718_m1; *Periostin/Postn* Mm01284919_m1; *SerpinB2* Mm00440905_m1; *Cxcl2* Mm00436450_m1; *Csf3* Mm00438334_m1; *Egfr* Mm01187858_m1;*, Erbb2* Mm00658541_m1; *Erbb3* Mm01159990_g1 *Areg* Mm01354339_m1; *Btc* Mm00432137_m1; *Egf* Mm00438696_m1; *Ereg* Mm00514794_m1*; Hbegf* Mm00439306_m1, *Tgfa* Mm00446232_m1; (all from Life Technologies) and Gapdh (PrimerDesign). mRNAs were measured by reverse transcription quantitative PCR. Relative mRNA expression was analysed using the ΔΔCt method^28^ with *Gapdh* as housekeeping gene*.*

### 2.6 Immunohistochemistry

The lungs were inflation‐fixed at constant pressure with 10% neutral buffered formalin and embedded in paraffin wax. 5μm sections were stained using haematoxylin and eosin (H&E).

### 2.7 Cytokine measurements

IL‐13, KC, CCL11, CXCL2 and CSF3 proteins were measured by ELISA (R&D Systems) according to the manufacturer's instructions.

### 2.8 Statistical analyses

Normal distribution of the numeric data was evaluated, and appropriate parametric or non‐parametric statistical tests applied. Parametric data are plotted as mean with one standard deviation (SD) while non‐parametric data are shown as boxes representing the 25 and 75% interquartile ranges, whiskers depicting the minimum and maximum values. Statistical significance was assessed by using Student's *t* test (parametric, unpaired data) with Welch's correction if SDs were not equal or Mann‐Whitney test (non‐parametric, unpaired data) for comparisons between two groups. For comparison of three or more groups, a one‐way ANOVA with Dunn's multiple comparison test (parametric data) or Kruskal‐Wallis test with Dunn's test for correction for multiple comparisons (non‐parametric data) was used. For comparison of two or more groups with two independent variables, a two‐way ANOVA with Tukey's multiple comparison test was used. * = *P* < .05, ** = *P* < .01, *** = *P* < .001.

### 2.9 The U‐BIOPRED cohort

U‐BIOPRED is a multi‐centre study that enrolled 311 severe, non‐smoker asthmatics, 88 mild/moderate non‐smoking asthmatics and 101 healthy controls.^8^ Among these participants, 61 severe asthmatics, 36 mild/moderate asthmatics and 44 healthy volunteers underwent fiberoptic bronchoscopy and epithelial brushing. Epithelial brushings were processed into RNAlater for subsequent analysis on Affymetrix U133 Plus 2.0 microarrays, and only those passing stringent quality control were analysed.^10^ All participants provided written informed consent to participate in the study which was approved by national ethics committees. The transcriptomic data are stored as GSE76226.^29^

Using this dataset, epithelial expression of EGFR and EGFR ligands was compared between groups using SPSS v24. Paired *t* tests were applied to the log2‐transformed transcriptomic data, while the clinical data were analysed by Kruskal‐Wallis test and Mann‐Whitney *U* or Student *t* tests depending on the type of data distribution; *P* < .05 was considered significant. Topological data analysis (TDA) was then applied to the epithelial transcriptomic data as described previously,^30^, ^31^ with some modification, using the Ayasdi Core software (Ayasdi, Menlo Park, CA) with a norm correlation metric and the neighbourhood lens (resolution, 20 bins; gain, 4.00). Clinical and pathobiological data were then overlaid as metadata onto the generated TDA network to look for associations.

## 3 RESULTS

### 3.1 IL‐13 induces BHR and a mixed inflammatory phenotype

**Figure 1:**
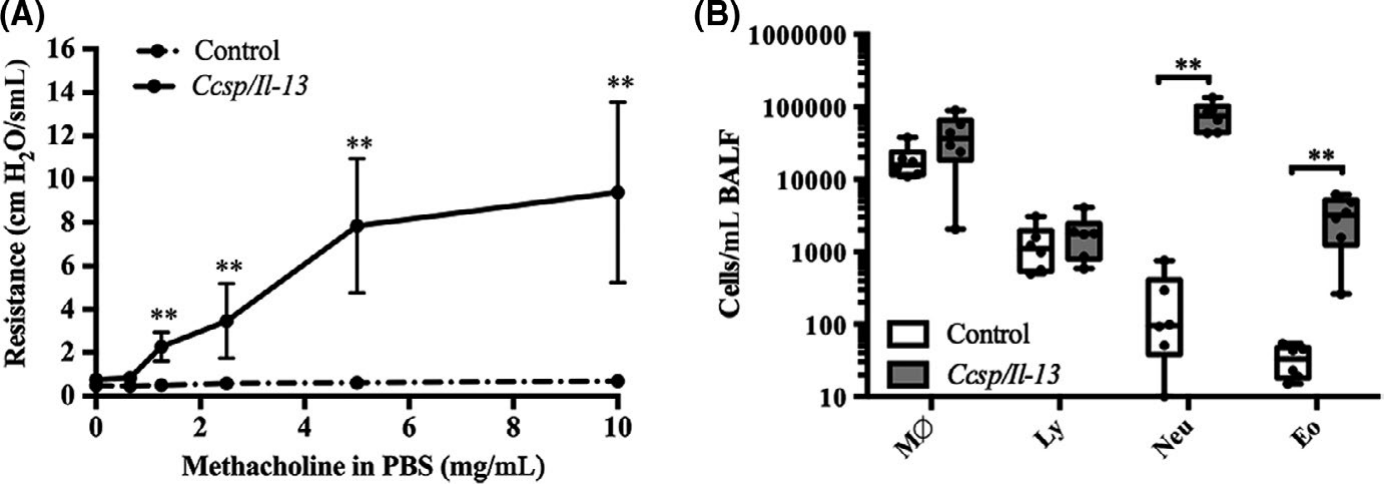
IL‐13 induces BHR and mixed inflammatory cell airway inflammation. A, Bronchial hyperresponsiveness to methacholine challenge of control and *Ccsp/Il13* transgenic mice on DOX for 7 d. Resistance (R: cmH_2_O.s/mL) at methacholine concentrations from 0 to 10 mg/mL in phosphate‐buffered saline (PBS). Data are shown as mean ± SEM. B, Differential cell counts of BALF from *Ccsp/Il13* and control mice (M∅ = macrophages; Ly = lymphocytes; Neu = neutrophils; and Eo = eosinophils). Box plots show medians and 25th to 75th percentiles, and whiskers represent minimum and maximum values; all data points are shown. N = 6 mice per group. Data are representative of three independent experiments. Statistical analysis was performed using two‐way ANOVA with Tukey's multiple comparison test. **P < .01

IL‐13 was significantly elevated (Figure S1A, B) when *Ccsp/Il13* mice were fed DOX to induce transgene expression. As previously reported,^25^ when challenged with methacholine, these IL‐13–expressing mice showed a significant increase in BHR (*P* < .002) (Figure 1A); furthermore, both eosinophils and neutrophils were significantly elevated in BALF (Figure 1B) compared to single transgenic littermate controls. Histochemical analysis of lung sections also demonstrated a significant increase in airway inflammation, as well as goblet cell metaplasia (Figure S2A,B) accompanied by *Mucin 5AC (Muc5ac)* mRNA expression (Figure S2C).

### 3.2 Both “type‐2” and “‐type‐17” biomarkers are induced by IL‐13

**Figure 2:**
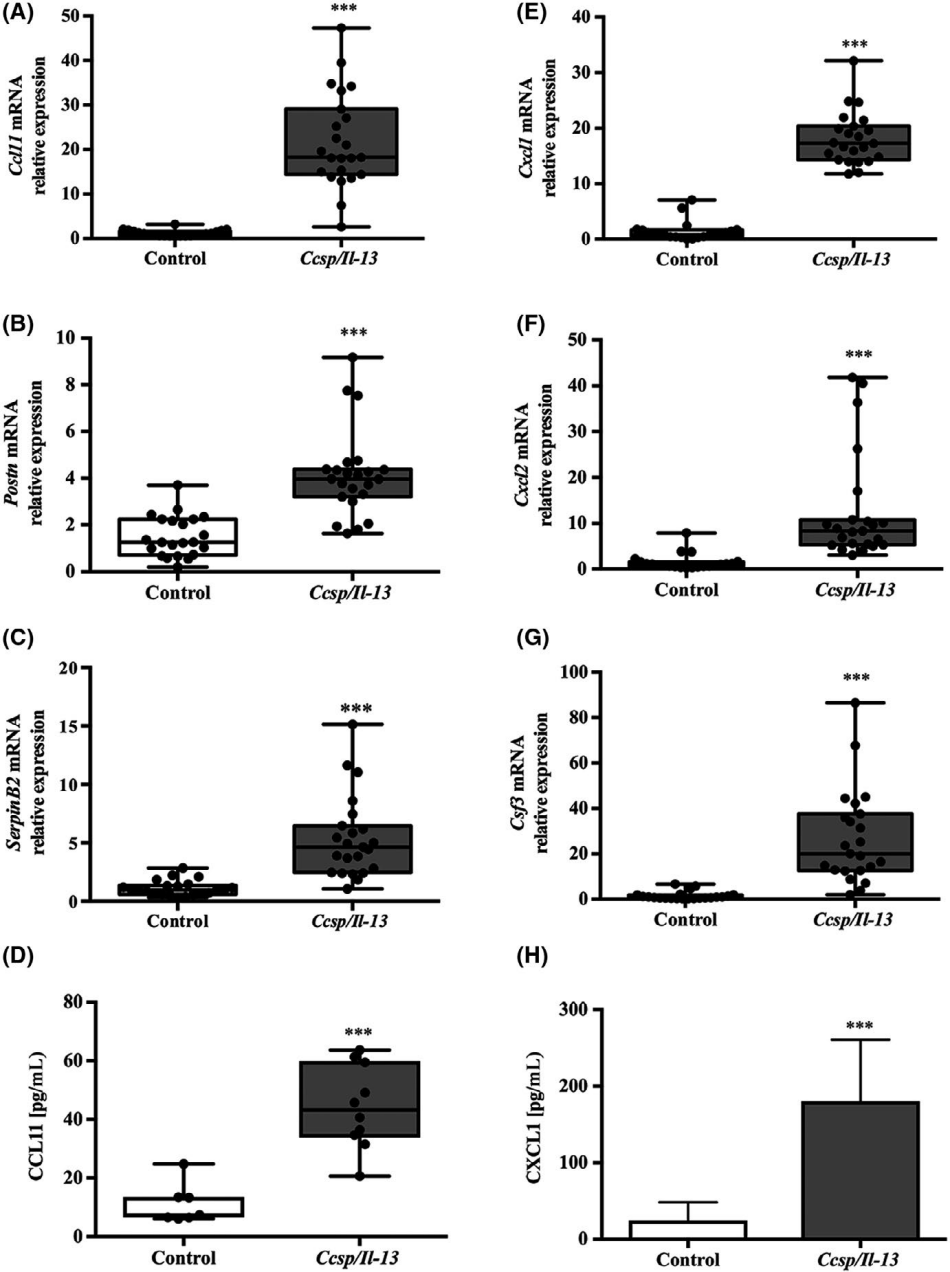
Expression of IL‐13 induces expression of both type‐2 and pro‐neutrophilic mediated markers. Relative mRNA expression in whole‐lung lobe lysates from littermate controls (white bars) vs* Ccsp/Il13* mice (grey bars) after induction of IL‐13 for 7 d for A, *Ccl11,* B, *Postn*, C, *Serpinb2*, E, *Cxcl1/KC*, F, *Cxcl2* and G, *Csf3.* ELISAs for D, CCL11 and H, CXCL1 protein levels in BALF. For mRNA expression, n = 22 for controls and n = 23 for *Ccsp/Il13* mice and n = 7 and n = 10, respectively, for protein, from three independent experiments. Non‐parametric data are shown as box plots with medians and 25th to 75th percentiles, and whiskers representing minimum and maximum values with all data points shown; parametric data are shown as mean + SD. Statistical analyses were performed using Mann‐Whitney test or Student's *t* test. ***P < .001

Markers of “type‐2”–mediated inflammation including *eotaxin/Ccl11, periostin/Postn* and *SerpinB2* (Figure 2A‐C) mRNAs were all significantly elevated in lungs of IL‐13–expressing mice. Expression of mRNAs for genes including *chemokine (C‐X‐C motif) ligand 1* (*Cxcl1/Kc), Cxcl2* and *Colony Stimulating Factor 3 (Csf3)* (Figure 2E‐G) which are more usually associated with “type‐17” responses was also increased by IL‐13; however, expression of *Il‐17a* mRNA was undetectable in the lungs of IL‐13 expressing mice (*C*_t_ values > 38)*.* ELISAs for CCL11 and CXCL1/KC confirmed elevation of these cytokines in BALF of IL‐13–expressing mice (Figure 2D,H). BALF CCL11 protein levels were significantly correlated with eosinophil numbers (*r* ^2^ = 0.74, *P* = .02) (Figure S3A) but not neutrophil numbers, whereas CXCL1 levels were strongly correlated with neutrophil numbers (*r* ^2^ = 0.9, *P* = .003) (Figure S3B) but not with eosinophil numbers. Furthermore, BALF CXCL1 levels were correlated with the amount of IL‐13 in the BALF (*r* ^2^ = 0.74, *P* = .001) (Figure S3C) consistent with a role for IL‐13 in driving airway neutrophilia via CXCL1. CXCL2 protein was also significantly elevated in both the BALF and lung lysate (Figure S4A,B).

### 3.3 Corticosteroid treatment does not influence neutrophilic inflammation and BHR

**Figure 3:**
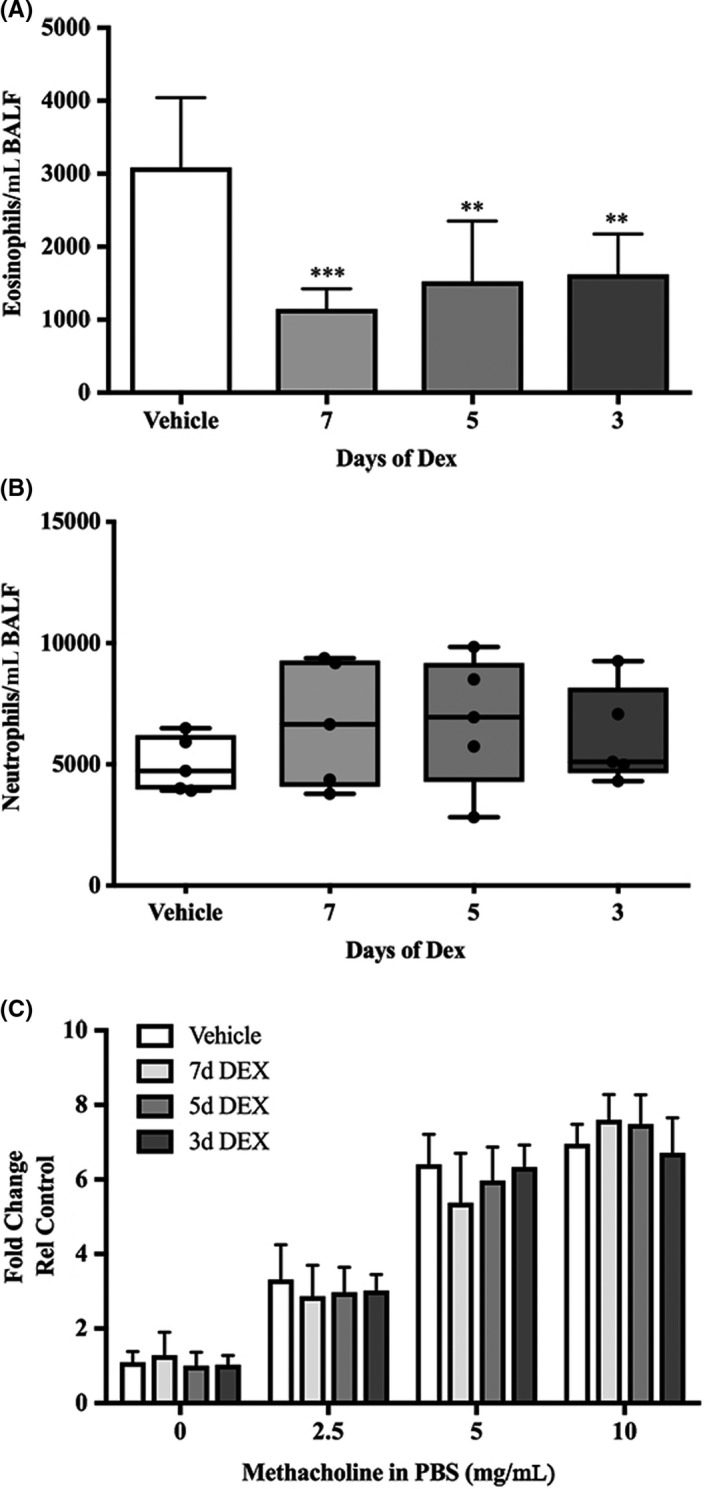
Dexamethasone reduces eosinophil count but does not affect neutrophils or suppress BHR. Differential inflammatory cell counts for A, Eo, eosinophils and B, Neu, neutrophils in BALF after a time‐course Dex treatment (grey bars) vs sham treatment (white bars) in *Ccsp/Il13* mice*.* C, Airway resistance in response to increasing concentrations of methacholine (0‐10 mg/mL). Non‐parametric data are shown as box plots with medians and 25th to 75th percentiles, and whiskers representing minimum and maximum values with all data points shown; parametric data are shown as mean + SD. N = 5 per group from two independent experiments. Statistical analysis was performed using one‐way ANOVA or Kruskal‐Wallis test with Dunn's test for correction for multiple comparisons. **P < .01, ***P < .001

To determine how airway responses are modulated by corticosteroid treatment, IL‐13 was induced in *Ccsp/Il13* mice for 7 days and dexamethasone (Dex) was given via intraperitoneal injection for the duration of IL‐13 induction (7 days) or for the final 5 or 3 days of induction. The control group received DOX to induce IL‐13 and were sham‐treated with saline for 7 days. Dex significantly reduced eosinophil numbers after 7 days of treatment; furthermore, even shorter treatments given during the final 3 or 5 days of IL‐13 induction significantly suppressed eosinophil numbers (Figure 3A). In contrast, neither infiltration of neutrophils (Figure 3B) nor BHR (Figure 3C) were affected by presence or duration of Dex treatment. Although long‐term treatment with corticosteroids can have metabolic effects,^32^ the dose and duration used had no effect on body weight (Figure S5). In addition, Dex treatment did not cause any inflammation in the airways of similarly treated sTg mice, nor did it affect body weight.

### 3.4 Corticosteroids dampen “type 2,” but not pro‐neutrophilic cytokine responses

**Figure 4:**
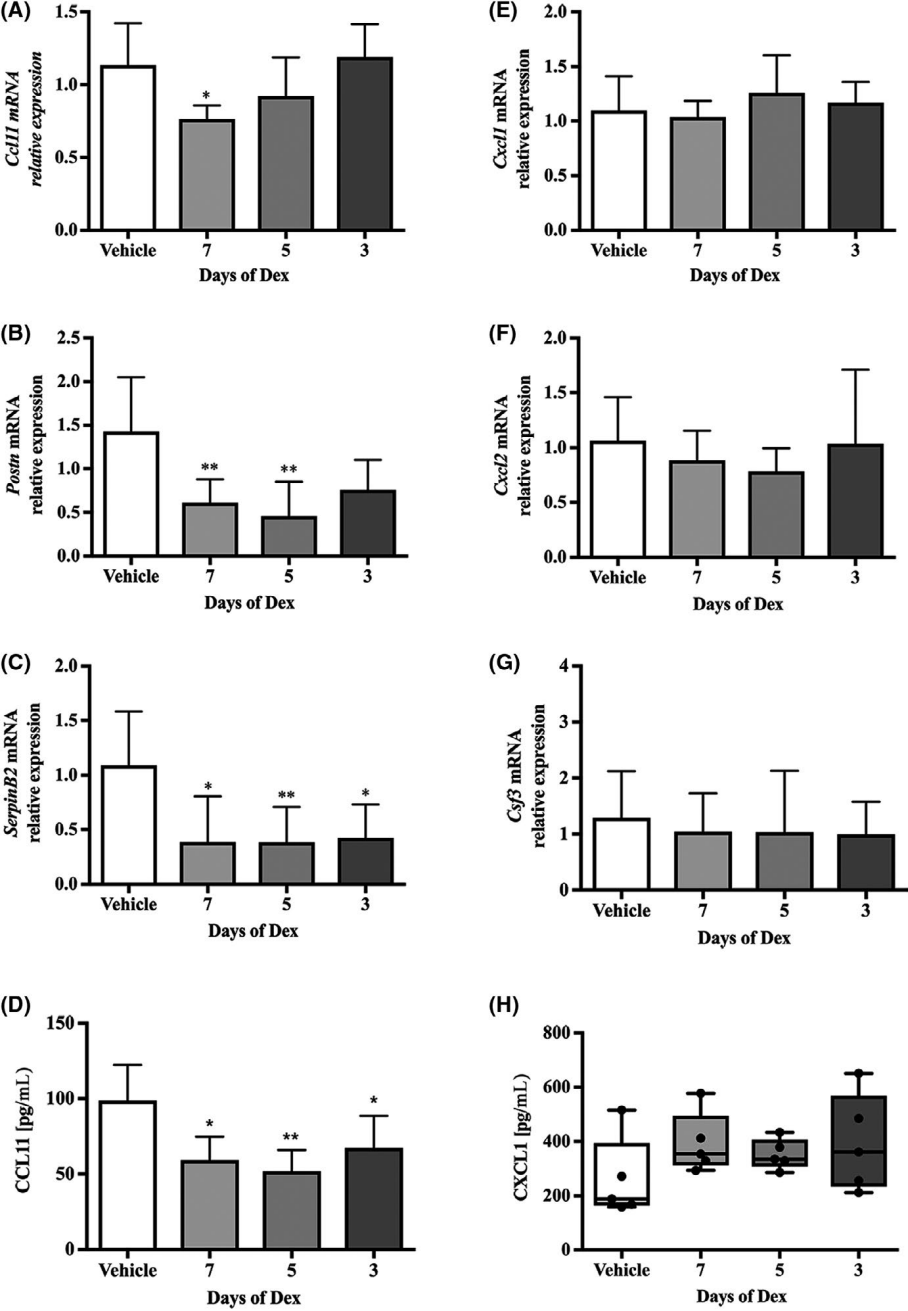
The type 2 lung inflammation signature is reduced by dexamethasone, but not the pro‐neutrophilic responses. Relative mRNA expression in whole‐lung lobe lysates from *Ccsp/Il13* mice after induction of IL‐13 for 7 days and dexamethasone treatment for all 7, the final 5 or 3 days (grey bars) (n = 5) vs vehicle‐treated *Ccsp/Il13* mice (white bars) (n = 5). A, *Ccl11,* B, *Postn,* C, *Serpinb2,* E, *Cxcl1/KC,* F, *Cxcl2* and G, *Csf3.* ELISAs for D, CCL11 and H, CXCL1 protein levels in BALF. Parametric data are expressed as mean + SD and non‐parametric data as box plots showing medians and 25th to 75th percentiles, and whiskers representing minimum and maximum values with all data points shown. Data are from two independent experiments. Statistical analysis was performed using one‐way ANOVA or Kruskal‐Wallis test with Dunn's test with correction for multiple comparisons. *P < .05, **P < .01

Analysis of the effects of Dex on pulmonary gene expression revealed that *Ccl11, Postn* and *SerpinB2* mRNAs were each suppressed by Dex treatment (Figure 4A‐C) and a significant suppression in CCL11 protein release was evident after only 3 days of Dex treatment (Figure 4D). Changes in *Muc5ac* mRNA were also evident after 7 days of corticosteroid treatment (Figure S6). In contrast, *Cxcl1/Kc, Cxcl2* and *Csf3* remained unchanged by Dex, regardless of duration of treatment (Figure 4E‐G). Similarly, protein levels of CXCL1/KC (Figure 4H) and CXCL2 in either BALF or lung lysate were unaffected by Dex treatment (data not shown).

### 3.5 The EGFR is a mediator of IL‐13–induced corticosteroid‐insensitive responses in vitro and in vivo

We have previously reported that EGFR activation drives corticosteroid‐insensitive release of IL‐8 from human bronchial epithelial cells.^33^, ^34^ As IL‐13 has been shown to drive epithelial proliferation via an EGFR/TGF‐α autocrine loop,^35^ we explored whether EGFR activation could contribute to the responses observed in the IL‐13 mice. Following 3Rs principles, we initially pursued in vitro mechanistic studies using cultures of murine tracheal epithelial cells (mTECs) from control and *Ccsp/Il13* mice. Initial characterization showed that, regardless of genotype, the cultures developed a TER > 1000 Ω.cm^2^ (Figure S7). When DOX was applied to the cultures, it induced IL‐13 mRNA expression and IL‐13 protein release only in the *Ccsp/Il*13 mTECs (Figure S8A‐C) and this caused a significant drop in TER at 24h, consistent with the known effect of IL‐13 on ionic permeability 36 (Figure S8D). Induction of IL‐13 also resulted in an increase in *Ccl11* mRNA expression which was suppressed by Dex (Figure S9).

**Figure 5:**
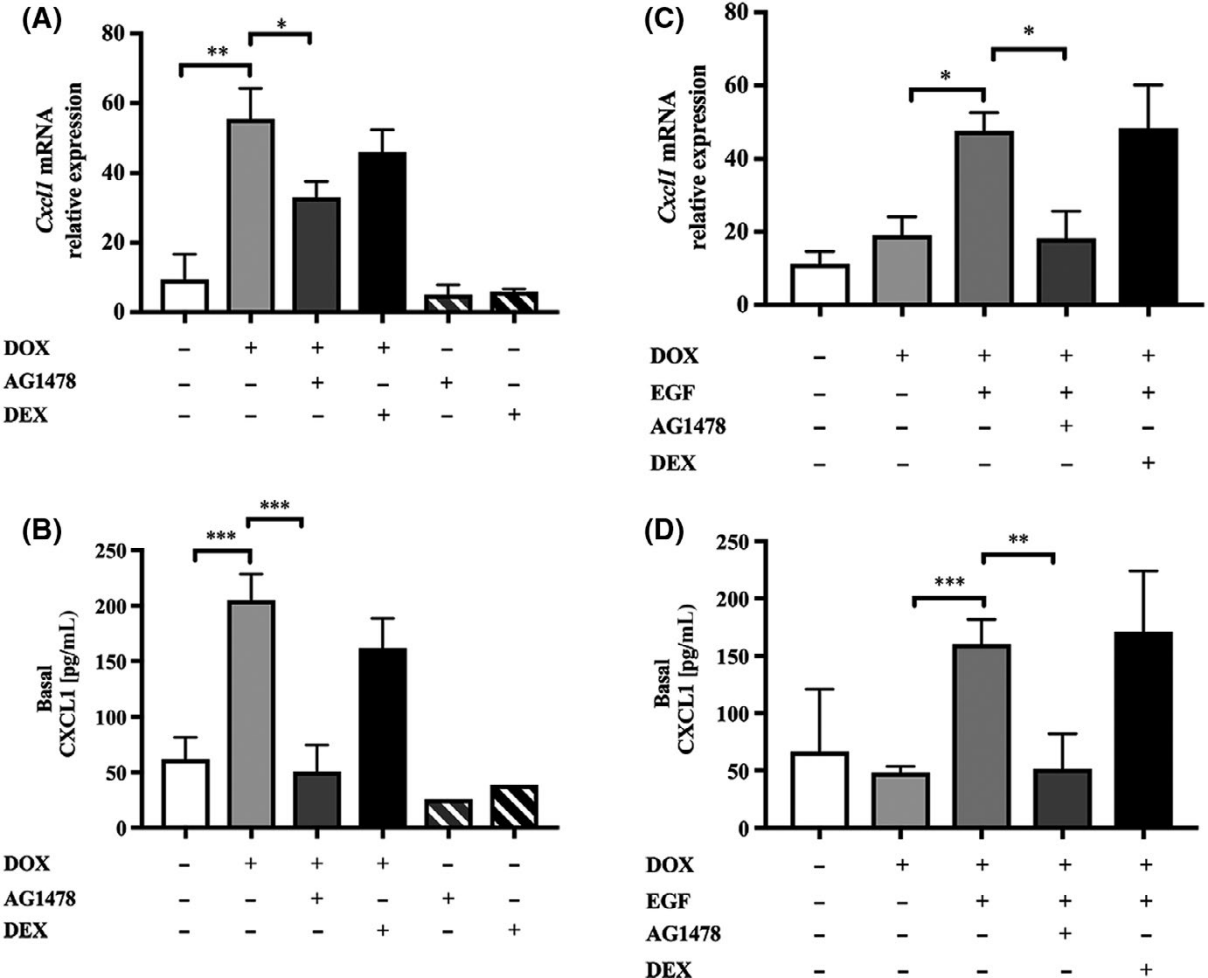
IL‐13 induces expression of *Cxcl1*/CXCL1 that can be blocked by the EGFR inhibitor AG1478 in mTEC cultures. A, Relative *Cxcl1* mRNA expression and B, CXCL1 protein expression in *Ccsp/Il13* mTECS treated, where indicated, with DOX (to induce IL‐13), AG1478 or Dex for 72 h. C, D, Relative *Cxcl1*/CXCL1 mRNA and protein expression, respectively, in control mTECs treated, where indicated, with DOX, EGF, AG1478 or Dex. Data are expressed as mean + SD. Experiments were performed in duplicate and are from three independent experiments. Statistical analysis was performed using one‐way ANOVA with Dunn's multiple comparison. *P < .05, **P < .01, ***P < .001

Having validated the model, we evaluated the effect of IL‐13 on induction of the pro‐neutrophilic cytokine genes and compared the inhibitory effect of Dex or the highly selective EGFR tyrosine kinase inhibitor, AG1478.^37^ Expression of *Cxcl1/Kc* mRNA was significantly increased when IL‐13 was induced with DOX (Figure 5A) and this was accompanied by a fourfold induction of basolateral CXCL1/KC protein secretion (Figure 5B). In the control mTECs, DOX treatment failed to induce *Cxcl1/Kc* mRNA or CXCL1/KC protein; however in the presence of EGF, *Cxcl1/*CXCL1 expression was increased (Figure 5C,D), consistent with a role for the EGFR in driving expression of this neutrophilic chemokine. As observed in vivo, Dex had no significant effect on *Cxcl1/Kc* mRNA or CXCL1/KC protein release from the IL‐13–expressing cultures (Figure 5A,B). Similarly, EGF‐induced release of CXCL1/KC protein was insensitive to inhibition by Dex (Figure 5C,D). In contrast, the EGFR inhibitor AG1478 significantly reduced both apical and basolateral release of CXCL1/KC from IL‐13‐ or EGF‐stimulated cells (Figure 5B,D). Similar results were obtained for *Cxcl2*/CXCL2 mRNA and protein expression induced by IL‐13 expression or by EGF treatment of cells from control mice (Figure S10A‐D): these were not affected by Dex, but were suppressed by the EGFR inhibitor.

**Figure 6:**
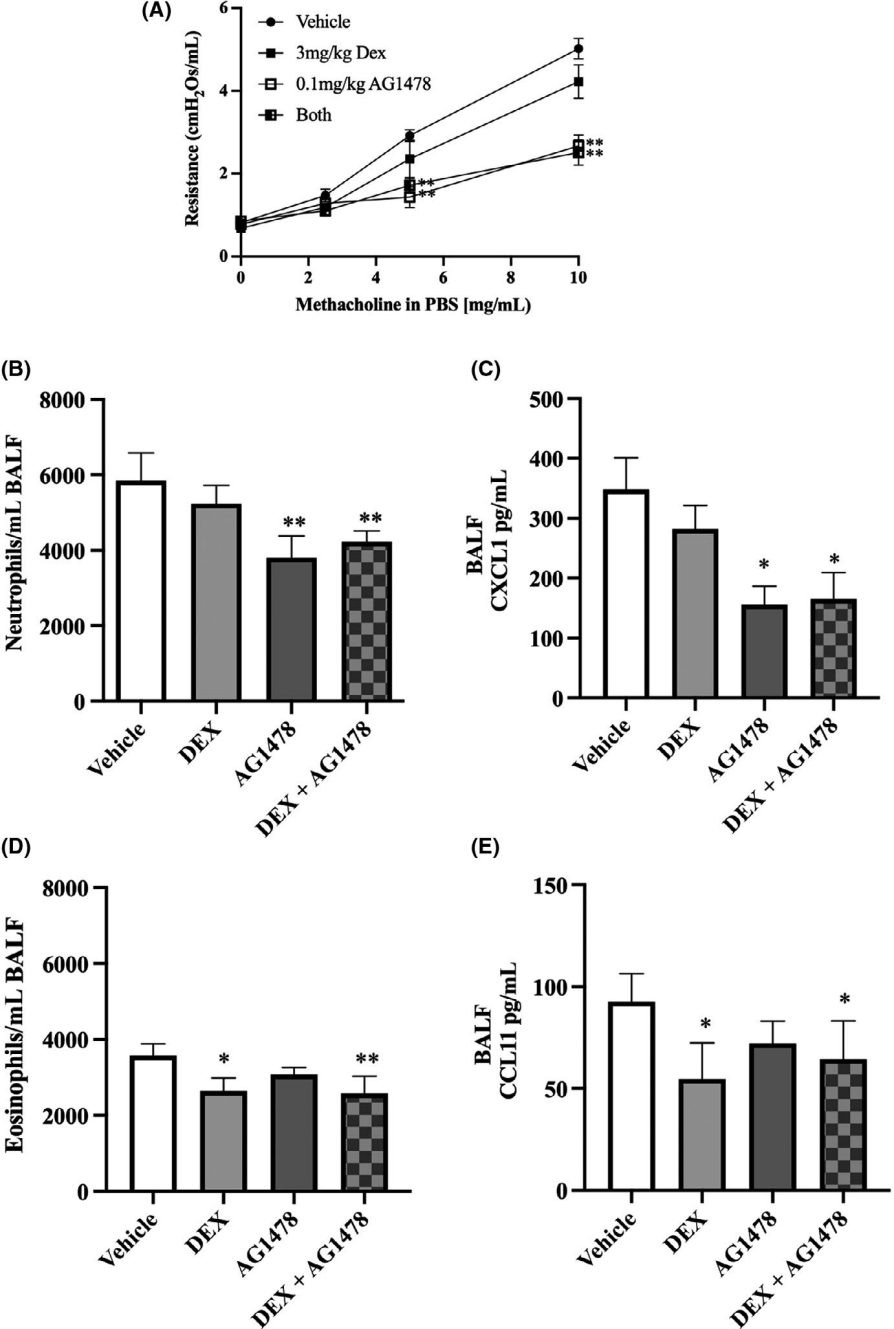
The EGFR inhibitor AG1478 blocks the pro‐neutrophilic responses caused by IL‐13 expression in vivo*. Ccsp/Il13* mice were fed DOX for 7 d to induce IL‐13 and treated with dexamethasone (light grey bars), AG1478 (dark grey bars), dexamethasone + AG1478 (hatched bars) or vehicle control (white bars). A, Airway resistance in response to increasing concentrations of methacholine (0‐10 mg/mL). B and D, Differential inflammatory cell counts in BALF for neutrophils and eosinophils, respectively. C and E, CXCL1 or CCL11 protein expression, respectively, measured by ELISA in whole‐lung lobe lysates; data are shown as mean + SD. N = 6 per group from two independent experiments. Statistical analysis was performed using one‐way ANOVA. *P < .05, **P < .01

Based on our in vitro findings, we investigated whether EGFR inhibition in vivo had the ability to modulate the corticosteroid‐refractory responses observed in the *Ccsp/Il13* transgenic mouse model. Thus, IL‐13 was induced with DOX and the animals were treated with AG1478 and/or Dex for 7 days. This revealed that AG1478 was able to suppress BHR, airway neutrophilia and pro‐neutrophilic cytokine expression (Figure 6A‐C), while it had no significant effect on airway eosinophilia and type‐2 biomarker expression (Figure 6D,E). In contrast, Dex while significantly suppressed the type 2 responses (eosinophils and CCL11 release), the combination of AG1478 and Dex significantly suppressed all response**s**.

### 3.6 Transcriptomic analysis of U‐BIOPRED cohort

**Figure 7:**
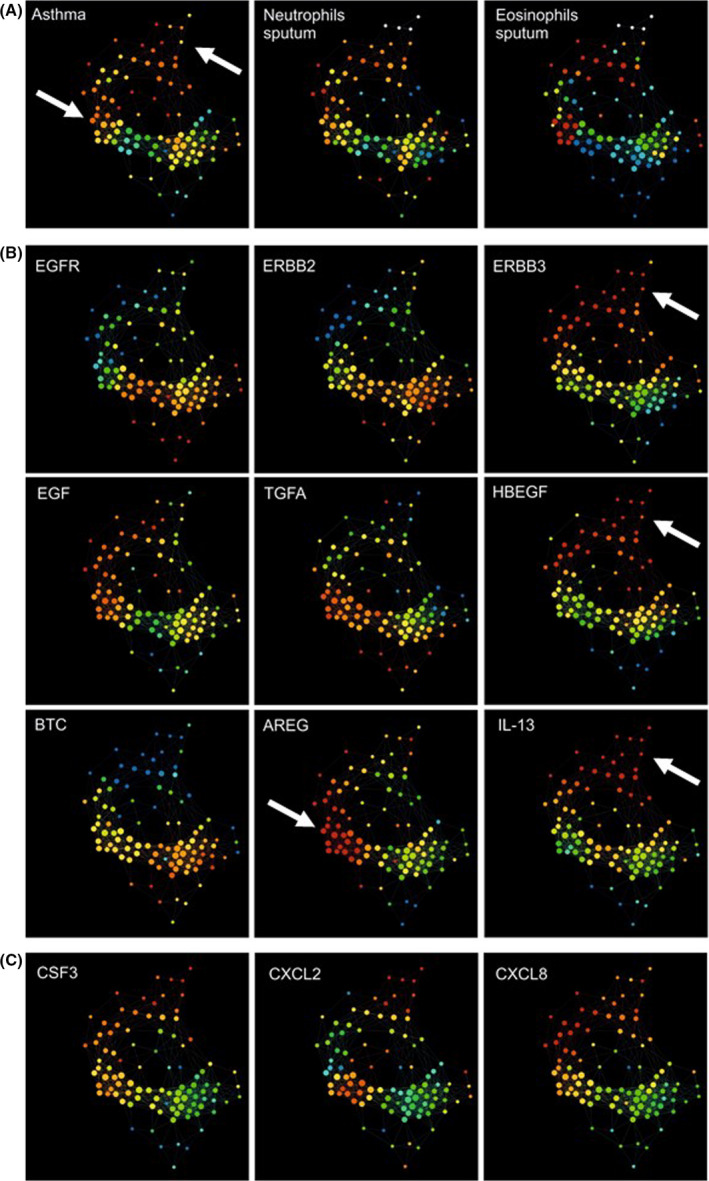
Topological data analysis of gene expression obtained from bronchial brushings from the U‐BIOPRED cohort. A TDA network was constructed using gene expression data obtained from bronchial brushings from non‐smoking severe asthmatics (n = 61), mild‐to‐moderate asthmatics (n = 36) and healthy controls (n = 44). As indicated, metadata were then applied for (A) asthma severity, sputum neutrophils or eosinophil counts; (B) *EGFR*, *ERBB2*, *ERBB3,* EGFR ligands or *IL13*; and (C) *CSF3, CXCL2* or *CXCL8*. Nodes are coloured by intensity from blue (low) to red (high). Arrows point to the regions of interest referred to in the Results sections

To explore the relevance of our findings in human asthma, epithelial transcriptomic data from healthy (n = 44), mild‐to‐moderate asthma (n = 36) and severe asthma (n = 61) were clustered by TDA which provides a general framework to analyse high dimensional data in a manner that is insensitive to the particular metric chosen and provides dimensionality reduction and robustness to noise.^31^, ^38^ It has the advantage over standard clustering methodologies in that it provides geometric representations of multi‐dimensional data and it is often possible to find subgroups in data sets that traditional methodologies fail to find. The clinical, pathobiological data and log2‐transformed expression levels of *ERBB* receptors and EGFR ligands were then applied as metadata (Figure 7A,B). This revealed a cluster of severe asthmatics with neutrophilia and varying numbers of eosinophils (Figure 7A). While *EGFR* expression was down‐regulated in this cluster, *ERBB3* and several EGFR ligands, including heparin‐binding EGF *(HB‐EGF)*, epiregulin *(EREG)* and *EGF,* were up‐regulated (Figure 7B). This severe asthma cluster was associated with increased expression of *CSF3, CXCL2* and *CXCL8*, genes usually associated with an “IL‐17 signature”,^39^ although it was evident that expression of these genes was heterogeneous across the cluster. Furthermore, within this severe asthma cluster it was possible to identify two sub‐clusters: one was characterized by highest *IL13* (Figure 7C), *ERBB3* and *HB‐EGF* expression; the other sub‐cluster was characterized by highest amphiregulin (*AREG)* expression suggesting that there may be distinct sub‐phenotypes of severe asthma that can be defined by different *ERBB* receptor and ligand combinations. Further analysis using previously defined genes associated with the IL‐13 or IL‐17 signatures^18^ showed that *EGFR* and *ERBB2* were significantly down‐regulated and *ERBB3* up‐regulated in severe asthmatics with “IL‐13” or “IL‐17 high” phenotypes but not in those subjects with an “IL‐13/IL‐17 low” phenotype. In contrast, in the latter group, *ERBB4* was the only *ERBB* family member to show significant modulation (Table 1). For the EGFR ligands, *HBEGF* was significantly increased in the “IL‐13 high” group whereas *AREG, EGF* and *EREG* were significantly up‐regulated in the “IL‐17 high” group.

**Table 1:** Comparison of ERBB receptor and ligand expression in “IL‐13 high” and “IL‐17 high” asthma clusters relative to health in the U‐BIOPRED cohort

n	“IL‐13 low/IL‐17 low”		“IL‐17 high”		“IL‐13 high”	
	53		22		9	
	Log2 fold change	*P*	Log2 fold change	*P*	Log2 fold change	*P*
*EGFR*	−0.017	.1761	**−0.079**	**.0001**	**−0.097**	**.0011**
*ERBB2*	−0.007	.2059	**−0.031**	**.0006**	**−0.026**	**.0157**
*ERBB3*	0.034	.0953	**0.083**	**.0043**	**0.098**	**.0019**
*ERBB4*	**0.096**	**.0008**	−0.090	.0501	−0.059	.2681
*EGF*	0.007	.6470	**0.076**	**.0003**	0.049	.0621
*TGFA*	0.006	.7253	−0.008	.7356	−0.027	.4088
*HBEGF*	0.015	.2612	0.027	.1056	**0.081**	**.0002**
*BTC*	−0.027	.1971	**−0.124**	**.0000**	**−0.071**	**.0359**
*AREG*	0.040	.3029	**0.225**	**.0011**	0.043	.5383
*EREG*	0.022	.5274	**0.344**	**.0019**	0.105	.0863

Note

Numbers are log2 fold change relative to expression in healthy participants; significant changes are highlighted in bold. The definition of “IL‐13 high” and “IL‐17 high” was based on that used in.^18^

**Figure 8:**
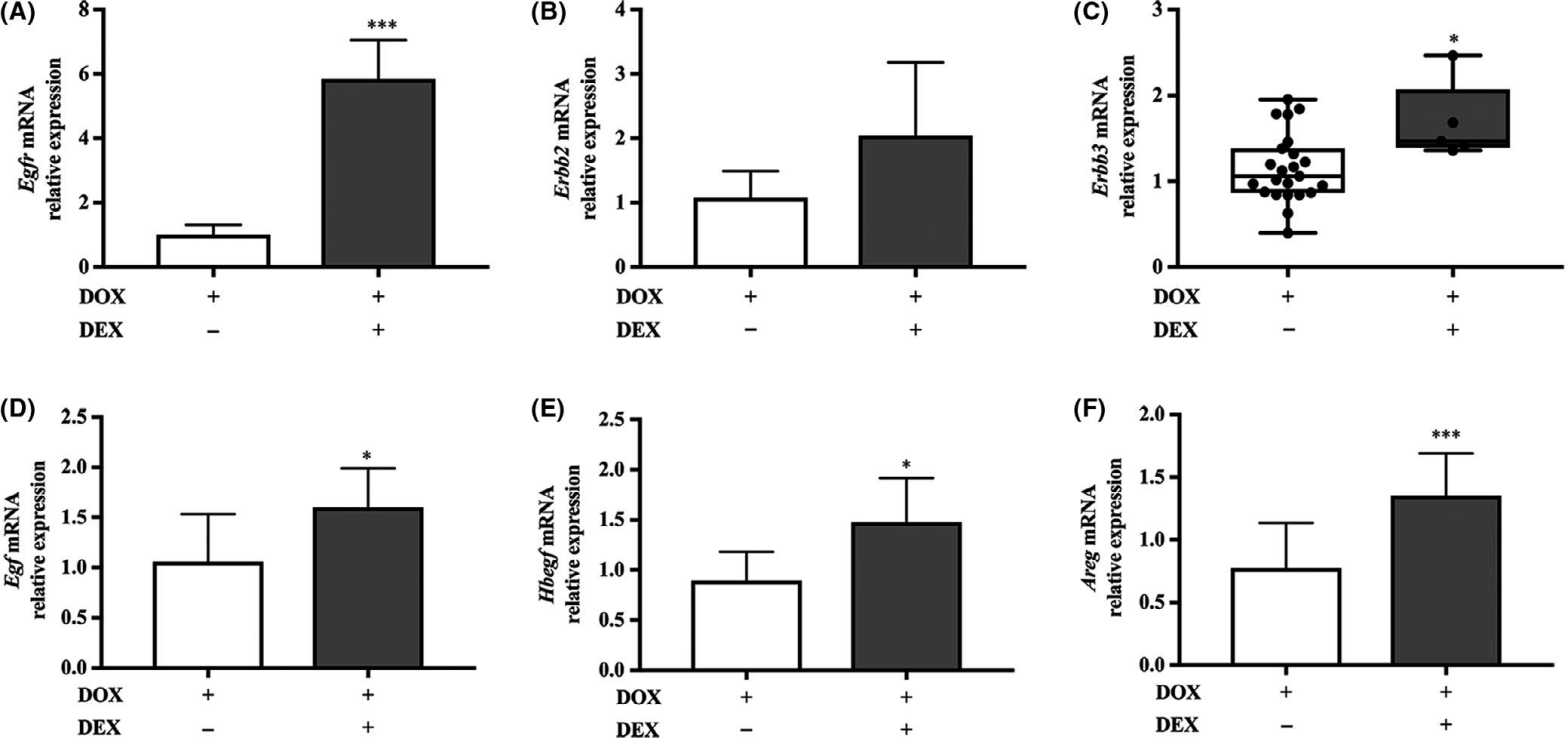
*Erbb* receptor and ligand mRNA expression in the IL‐13–expressing mouse model. Relative mRNA expression in whole‐lung lobe lysates from *Ccsp/Il13* mice after induction of IL‐13 for 7 d (white bars) (n = 23) vs concurrent dexamethasone (grey bars) treatment for 7 d (n = 6) for A, *Egfr*, B,* Erbb2*, C,* ErbB3* D, *Egf*, E, *Hbegf* and F, *Areg.* Parametric data are expressed as mean + SD and non‐parametric data as box plots showing medians and 25th to 75th percentiles, and whiskers representing minimum and maximum values with all data points shown. Data are from three independent experiments. Statistical analyses were performed using Student's *t* test or Mann‐Whitney test. *P<0.05, ***P < .001

The modulation of several *ERBB* receptors and ligands in human asthma led us to examine their expression in the mouse model. This revealed that corticosteroid treatment of the IL‐13–expressing mice increased expression of *Egfr*, *Erbb3*, *Egf, Hbegf* and *Areg,* whereas *Erbb2* was unaffected (Figure 8A‐F). As the low levels of EGFR in the U‐BIOPRED cluster appeared paradoxical, we postulated that EGFR activation may cause a negative feedback response to lower its mRNA expression. Therefore, we investigated whether this happened in mTEC cultures by studying IL‐13–induced *Muc5ac* expression in vitro. Under these conditions, IL‐13 stimulated *Muc5ac* in an EGFR‐dependent way (Figure S11A) and this was accompanied by suppression of *Egfr* mRNA expression (Figure S11B); since inhibition of EGFR activation with AG1478 prevented the suppression of *Egfr* mRNA (Figure S11B), these results are consistent with maintenance of EGFR signalling even when mRNA levels are decreased via a feedback loop.

## 4 DISCUSSION

Severe corticosteroid‐refractory asthma is a significant unmet medical. The disease is usually classified based on inflammatory cell profiles and related pathways, giving rise to the dichotomous definitions of “type‐2” and “non‐type 2” asthma with, by inference, distinct underlying mechanisms. In this study, we have shown that the lungs of transgenic mice expressing the classical type 2 pro‐allergic mediator, IL‐13, exhibited mixed eosinophilic and neutrophilic inflammation and increased expression of both type‐2 and non‐allergic, “type‐17” markers, even though *Il‐17* was not elevated in the lungs of the mice. We also found that the characteristic type‐2 biomarkers can be significantly suppressed (but not ablated) by corticosteroid treatment whereas BHR, neutrophilia and pro‐neutrophilic biomarkers were corticosteroid‐refractory. Through in vitro mechanistic studies, we demonstrated that these corticosteroid‐refractory IL‐13–induced pro‐neutrophilic responses were sensitive to inhibition of the EGFR and that in vivo inhibition of EGFR signalling in the IL‐13 mouse model suppressed pro‐neutrophilic cytokine expression and reduced airway neutrophilia and BHR. To relate these findings to human asthma, transcriptomic analysis of epithelial brushings from human volunteers identified a cluster of severe asthmatics displaying eosinophilia and neutrophilia with increased expression several EGFR ligands and *ERBB3* whose protein product is known to form heterodimers and signal with EGFR.^40^ Importantly, epithelial expression of *IL13* was found within a sub‐cluster of these asthmatic subjects, suggesting that our transgenic mouse model, which expresses IL‐13 in the airway epithelium, mirrors a sub‐phenotype of the human disease. Together, our data suggest that epithelial expression of the pro‐allergic cytokine, IL‐13, can drive corticosteroid‐resistant asthma which is mediated by epithelial EGFR/ERBB signalling driving a gene signature and phenotypic responses that are more classically associated with IL‐17 and Th17 immune responses. Furthermore, our data suggest that distinct “ype 17” sub‐phenotypes of severe asthma may arise through involvement of different ERBB receptor and ligand combinations.

A range of pathways have been implicated in the pathogenesis of corticosteroid‐refractory asthma, including increased activity of kinases, which phosphorylate the glucocorticoid receptor (GR) and prevent its nuclear translocation, and oxidative stress which inhibits the activity of histone deacetylase 2 (HDAC2) which mediates the actions of corticosteroids on pro‐inflammatory cytokine expression.^41^, ^42^ Other studies have suggested that the neutrophil‐high severe asthma phenotype which is poorly responsive to high‐dose corticosteroids^43^ might be a consequence of the treatment itself, since corticosteroids promote neutrophil survival.^44^, ^45^ Other studies have associated the neutrophilic phenotype with bacterial colonization or infection 46 and activation of IL‐17–producing helper T cells.^47^ However, in a randomized, double‐blind, placebo‐controlled study of brodalumab, a human anti‐IL‐17 receptor monoclonal antibody, in moderate‐to‐severe asthma there was no significant therapeutic effect.^48^ Thus, the mechanisms of corticosteroid‐refractory disease remain poorly understood, and as a result, no specific treatment is available for this difficult‐to‐treat group of patients.^42^

Previous studies have shown that IL‐13 can promote airway neutrophilia. For example, direct instillation of IL‐13 into the tracheae of rats results in neutrophilia driven by chemokines including IL‐8 49 while transgenic mice expressing IL‐13 have been shown to exhibit chronic inflammation involving both eosinophils and neutrophils, as well as lung remodelling.^25^, ^50^, ^51^ Of note, while eosinophilic inflammation caused by expression of the IL‐13 transgene diminished rapidly after removal of doxycycline, macrophages, neutrophils, lymphocytes and remodelling changes all persisted in the lung 3‐4 weeks after IL‐13 expression ceased. This was accompanied by sustained expression of genes, including multiple chemokines, that are likely involved in regulating the inflammatory responses initially stimulated by IL‐13.^25^ Consistent with this, we have demonstrated that IL‐13 drives pro‐neutrophilic “type 17” inflammatory gene and protein expression including *Cxcl1/Kc, Cxcl2* and *Csf3* and that amounts of CXCL1 were strongly correlated with both IL‐13 levels and neutrophil numbers in BALF. Beyond this, in the current study, we have identified that these responses, as well as BHR, are refractory to corticosteroid treatment. This contrasts with other murine models of allergic airway disease which are highly sensitive to inhibition by corticosteroids unless specifically manipulated by transfer of immune cells or by infection,^52^ but is similar to observations made in asthmatic patients who exhibit poor glucocorticoid responsiveness and have higher levels of serum IL‐8.^53^

We have previously shown that EGFR protein expression is increased in the bronchial epithelium of asthmatic patients according to disease severity^54^; furthermore, EGFR expression correlates with epithelial IL‐8 levels and the extent of neutrophilic inflammation in severe asthma.^33^, ^34^ Although involvement of the EGFR in inflammatory responses has been demonstrated previously using murine models of ovalbumin^55^ or house dust mite‐induced allergic inflammation,^56^, ^57^ both of these models are corticosteroid sensitive and so cannot address those corticosteroid‐refractory responses that are important in severe asthma. Using the IL‐13 transgenic mouse model, we identified a subset of IL‐13–driven corticosteroid‐refractory pro‐neutrophilic responses, and by culturing murine epithelial cell cultures in vitro, we identified that IL‐13 can induce similar pro‐neutrophilic cytokine responses that are refractory to corticosteroids, yet they can be suppressed by EGFR inhibition. We then confirmed these findings in the IL‐13 transgenic mouse model by showing that EGFR inhibition with AG1478 in vivo can prevent IL‐13–induced corticosteroid‐refractory pro‐neutrophilic inflammatory responses, airway neutrophilia and BHR. One key difference between the corticosteroid‐sensitive models of allergic airways inflammation and the transgenic mouse model is that *Il13* is expressed within the bronchial epithelium of the transgenic mouse, rather than in immune cells, as in the allergic models. As discussed below, analysis of the U‐BIOPRED cohort also identified *IL13* expression in the bronchial epithelium of a subgroup of severe asthmatic subjects, suggesting that the cellular provenance of IL‐13 may be an important determinant of disease activity.

The relevance of our findings in human asthma was explored using bronchial epithelial cell transcriptomic data from the U‐BIOPRED cohort. TDA analysis revealed that *EGFR* and *ERBB2* were significantly down‐regulated while *ERBB3* and a number of EGFR ligands, as well as *IL13*, were up‐regulated in severe asthmatics with mixed eosinophilic and neutrophilic inflammation, and this was accompanied by up‐regulation of *CSF3, CXCL2* and *CXCL8*.^39^ Although the low level of *EGFR* in this severe asthma cluster appears paradoxical, studies in cancer cells have shown that regulation of *EGFR* mRNA and protein expression is complex and can occur at multiple transcriptional and post‐transcriptional levels in a cell type–specific fashion.^58^, ^59^ In our in vitro studies, we showed that IL‐13–dependent EGFR activation suppressed *Egfr* mRNA expression suggesting feedback regulation of EGFR expression upon its activation in bronchial epithelial cells. As EGFR protein levels are increased in severe asthma,^54^ it would be of interest to study EGFR protein expression in the U‐BIOPRED cohort and to determine whether post‐translational mechanisms are also important for EGFR regulation.

A key finding from our analysis of the U‐BIOPRED data was that *IL13* mRNA expression was present in bronchial epithelial cells within a sub‐cluster of the severe asthma cluster where genes usually associated with an “IL‐17” gene signature^39^ were also up‐regulated. IL‐13 expression is normally associated with immune cells such as type 2 innate lymphoid cells (ILC2s), T helper 2 (Th2) cells mast cells and basophils, with ILC2s and Th2 cells being considered major sources of this cytokine,^60^ especially in type 2 asthma.^61^ However, induction of IL‐13 mRNA and protein has also been observed in wounded bronchial epithelial cells in vitro,^62^ and in our own unpublished work, we also have observed a significant increase in IL‐13 protein release from primary bronchial epithelial cells in response to challenge with double‐stranded RNA (data not shown). In the published work, release of IL‐13 following wounding was shown to enhance epithelial repair via HB‐EGF.^62^ Thus, it is significant that *IL13* expression in the U‐BIOPRED severe asthma cluster closely mirrored epithelial *HBEGF* expression suggesting a wound healing response in this subgroup of patients involving an IL‐13/HB‐EGF/EGFR axis. Of note, it has been shown that IL‐13 receptor α2 (IL‐13Rα2) can stimulate epithelial cell HB‐EGF production via TMEM219 and that TMEM 219 also contributes to optimal binding of IL‐13 to IL‐13Rα2.^63^ Unlike the Type II IL‐4 receptor complex which is a target Dupilumab 22 and binds both IL‐4 and IL‐13, IL‐13Rα2 is a high‐affinity receptor for IL‐13, but not IL‐4.^64^ Together, these observations may help to explain the so‐called “IL‐13 paradox”^65^ that while IL‐13 is involved in almost all aspects of asthma pathobiology, clinical trials using antibodies targeting IL‐13 have failed to demonstrate clinical benefit^66^ or any corticosteroid‐sparing effect.^67^ Thus, if IL‐13 is required for epithelial repair, potentially via IL‐13Rα2, neutralization of its effect on epithelial cells may prolong epithelial injury and impairment of epithelial barrier function, opposing any beneficial effects of the treatment on type 2 inflammation. Further work is required to investigate these possibilities.

In addition to the *IL13* sub‐cluster, we also identified a second sub‐cluster within the neutrophilic and eosinophilic severe asthma cluster which was characterized by high expression of *AREG*, *EGF* and *EREG.* Given that previous studies have identified an “IL‐17 high” severe asthma phenotype which exhibits expression of genes that are reported as altered in psoriasis lesions,^18^ it is significant that transgenic expression of AREG in murine skin causes a psoriasis phenotype characterized by marked epidermal hyperplasia, accompanied by neutrophilia and significantly increased CD4+ T cell infiltration.^68^, ^69^ Both AREG and HB‐EGF interact with heparan sulphate proteoglycans and with members of the tetraspanin family of membrane‐associated proteins which can regulate their distribution, bio‐availability and action on target cells and can also serve as cell surface co‐receptors, facilitating ligand‐receptor interactions.^70^ Thus, differential regulation of these two EGFR ligands offers the potential to fine‐tune their interaction with key target cells to drive distinctive EGFR/ERBB mediated responses that, in turn, may give rise to differing sub‐phenotypes of severe asthma. It is also noteworthy that the “IL‐13 low/IL‐17 low” severe asthmatic group (Table 1) was the only group to show a significant up‐regulation of *ERBB4,* whose function appears critically involved in reactions that affect cell fate.^71^ Its role within this group of severe asthmatics merits further investigation.

Based on the findings from the U‐BIOPRED cohort, further analysis of the IL‐13 expressing mouse model revealed that corticosteroid treatment caused significant modulation of the EGFR ligand family including *Hbegf* and *Areg*, as well as *Erbb3*. Unlike other family members, ERBB3 is considered “kinase dead” and requires heterodimerization with another family member such as EGFR or ERBB2 for phosphorylation; otherwise, it is refractory to ligand‐induced activation.^72^ Phosphorylated ERBB3 has multiple binding sites for phosphatidyl inositol‐3 kinase (PI3K)^73^ whose pathway is central to the development of BHR and inflammation.^74^ Furthermore, activation of PI3Kδ has been implicated in corticosteroid resistance as it causes Akt activation and inactivation of HDAC2, one of the mechanisms associated with corticosteroid resistance.^41^ In support of this suggestion, EGFR signalling is associated with increased PI3Kδ/Akt activation in ovalbumin‐induced airways inflammation.^55^ Our novel finding that ERBB3 is up‐regulated in severe asthma requires further investigation as it suggests that EGFR/ ERBB3 heterodimers may contribute to corticosteroid‐refractory asthma. As EGFR and corticosteroids also have beneficial effects on epithelial repair and barrier function,^54^ discriminating between pro‐inflammatory and pro‐repair functions for these pathways, and the role(s) of individual ligands and ERBB receptor heterodimers, should help to tailor more effective therapies for severe asthma.^45^ Such studies would offer the potential of exploiting the array of small molecule drugs and antibodies that have been developed for cancer therapy.^71^, ^75^

In summary, our study suggests that the prototypic type‐2 mediator, IL‐13, can give rise to both eosinophilic type‐2 and neutrophilic “type‐17” stereotypic responses, the latter being corticosteroid insensitive and mediated by epithelial EGFR signalling rather than classical immunological Th17 signalling. Based on our current findings, the IL‐13 transgenic mouse model should enable further understanding and dissection of the molecular pathways involved in corticosteroid‐refractory pathways, especially those involving EGFR/ERBB signalling in promotion of mixed granulocytic or neutrophilic inflammation and distinguishing them from beneficial pro‐repair pathways within the epithelium. This should enhance the development of new treatments that target this sub‐phenotype(s) of severe asthma.

## CONFLICT OF INTEREST

The authors declare the following conflicts of interest: DED and RD report personal fees from Synairgen, which is outside the submitted work. RD also reports receiving fees for lectures at symposia organized by Novartis, AstraZeneca and TEVA, consultation for TEVA and Novartis as member of advisory boards, and participation in a scientific discussion about asthma organized by GlaxoSmithKline. All are outside the submitted work. All other authors have nothing to declare.

## Supporting information

Figure S1

Figure S2

Figure S3

Figure S4

Figure S5

Figure S6

Figure S7

Figure S8

Figure S9

Figure S10

Figure S11

## Data Availability

The data that support the findings of this study are available either in the GEO repository (GSE76226) or can be obtained from the corresponding author upon reasonable request.
