# Supplementary figures and images for: Involvement of the epidermal growth factor receptor in IL‐13–mediated corticosteroid‐resistant airway inflammation
Source: Clin Exp Allergy. 2020 Mar 9;50(6):672–86. doi: 10.1111/cea.13591 (PMC7317751; doi:10.1111/cea.13591)

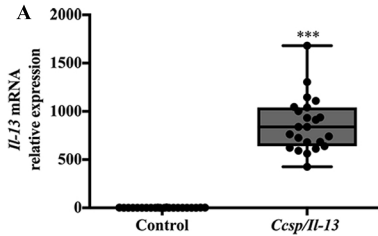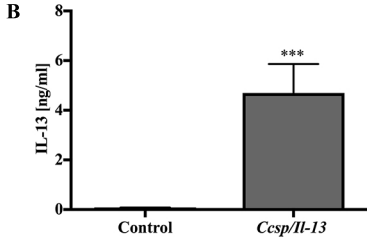

Figure S1

Supplement: Supplementary file 1 — Figure S1 [file CEA-50-672-s001.pdf]

**A**

Control

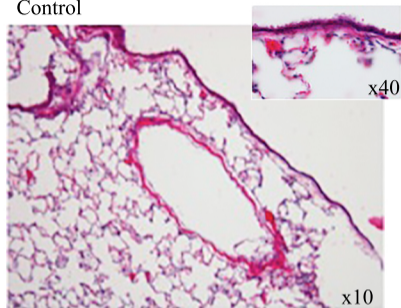**B***Ccsp/Ii-13*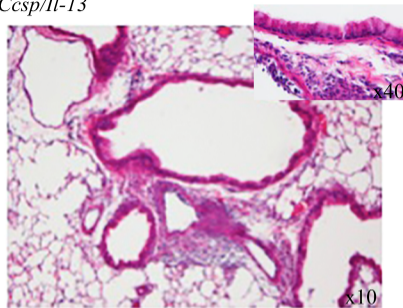**C**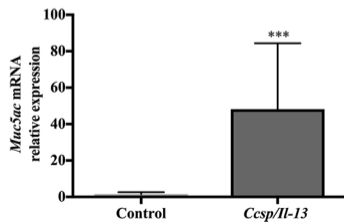

Figure S2

Supplement: Supplementary file 2 — Figure S2 [file CEA-50-672-s002.pdf]

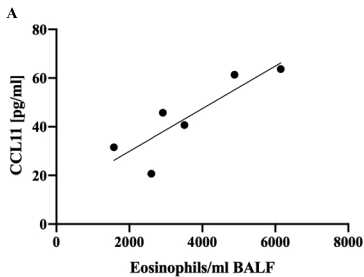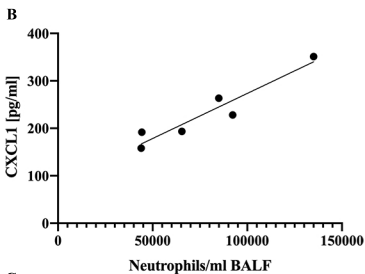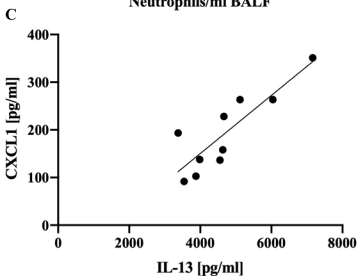

Figure S3

Supplement: Supplementary file 3 — Figure S3 [file CEA-50-672-s003.pdf]

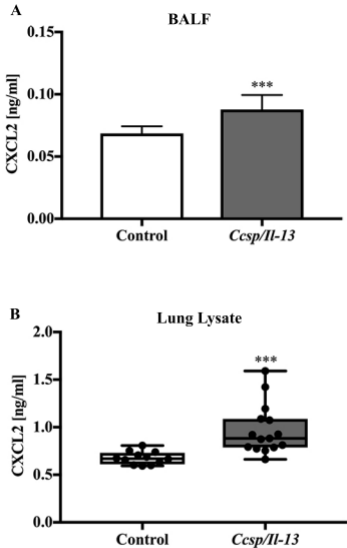

Figure S4

Supplement: Supplementary file 4 — Figure S4 [file CEA-50-672-s004.pdf]

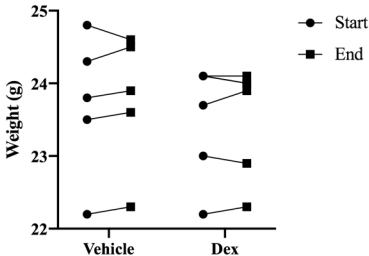

Figure S5

Supplement: Supplementary file 5 — Figure S5 [file CEA-50-672-s005.pdf]

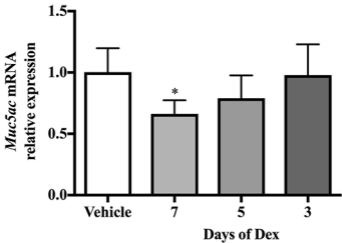

Figure S6

Supplement: Supplementary file 6 — Figure S6 [file CEA-50-672-s006.pdf]

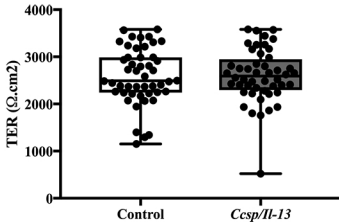

Figure S7

Supplement: Supplementary file 7 — Figure S7 [file CEA-50-672-s007.pdf]

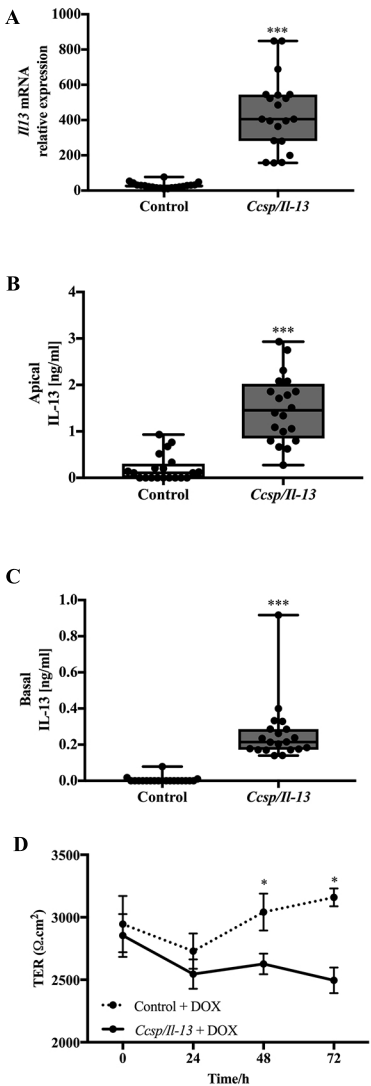

**Figure S8**

Supplement: Supplementary file 8 — Figure S8 [file CEA-50-672-s008.pdf]

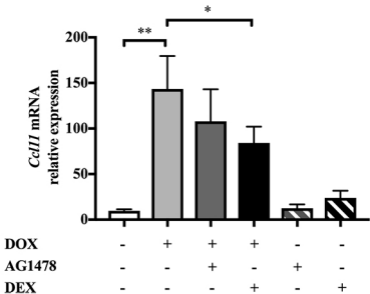

**Figure S9**

Supplement: Supplementary file 9 — Figure S9 [file CEA-50-672-s009.pdf]

**A**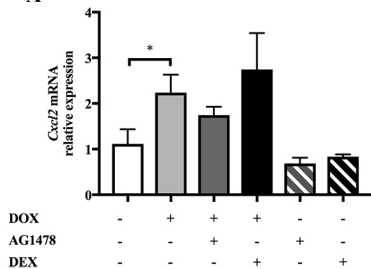**C**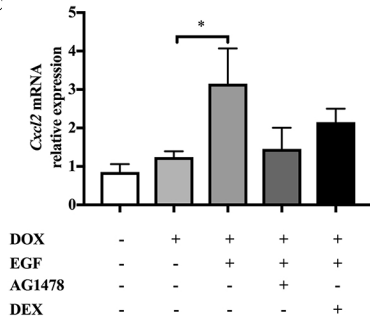**B**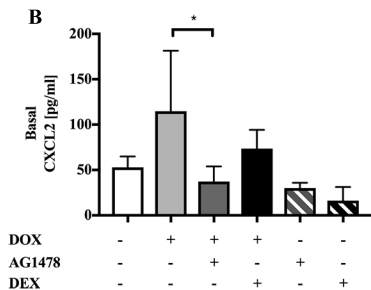**D**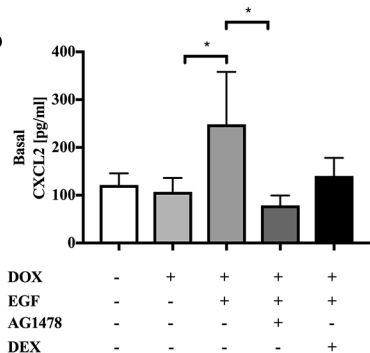

Supplement: Supplementary file 10 — Figure S10 [file CEA-50-672-s010.pdf]

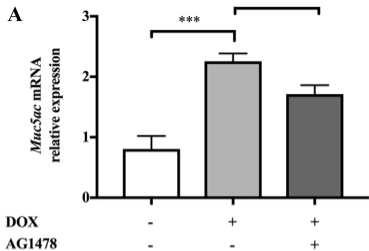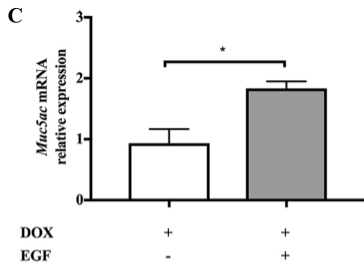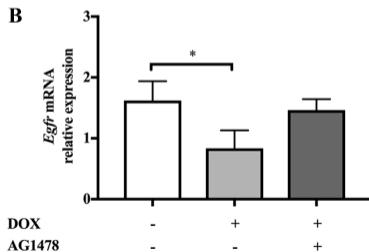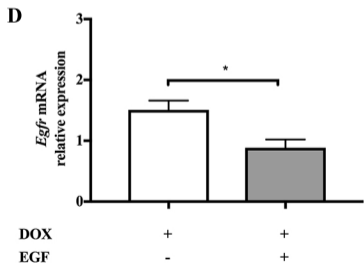

Figure S11

Supplement: Supplementary file 11 — Figure S11 [file CEA-50-672-s011.pdf]
